# Parental involvement in paediatric patient safety incidents in general practice: a cross-sectional study

**DOI:** 10.3399/BJGP.2024.0786

**Published:** 2025-08-12

**Authors:** Thomas Purchase, Isobel J McFadzean, Lauren Donovan, Jillian Beggs, Philippa Rees, Andrew Carson-Stevens

**Affiliations:** 1 Division of Population Medicine, Cardiff University, Cardiff, UK; 2 Population Policy and Practice, UCL, Great Ormond Street, Institute of Child Health, London, UK

**Keywords:** general practice, paediatrics, parents, patient safety

## Abstract

**Background:**

Children are a vulnerable patient group at risk of healthcare-associated harms, relying on others to support their healthcare needs. Parents, guardians, and caregivers may play a key role in both the aetiology and detection of unsafe care.

**Aim:**

To explore how and in what circumstances parents may inadvertently contribute to or help mitigate against paediatric patient safety incidents in general practice.

**Design and setting:**

A cross-sectional exploratory descriptive analysis was conducted of paediatric patient safety incidents occurring in general practice, with explicit evidence of parental involvement, between September 2014 and February 2023.

**Method:**

GPs coded the included reports’ free text using the PatIent SAfety (PISA) classification system to identify the types of incidents, contributing factors, or mitigatory actions, and the resultant harm outcomes. Coded data were described and summarised using frequency tables and cross-tabulations.

**Results:**

Of 374 reports included, most reports described mitigatory actions by parents (*n* = 287, 76.7%). Parents frequently mitigated incidents relating to medications (for example, prescribing), diagnosis and assessment, and administrative processes. Common mitigatory actions included recognising medication issues, chasing appointments, and providing feedback. These actions prevented harm or further harm from occurring in over half of reported incidents (54.4%, *n* = 156/287).

**Conclusion:**

The actions of parents have a direct impact on paediatric safety within general practice. This study identified several positive mitigatory actions taken by parents to keep their children safe. Primary care teams working to improve and design safer systems of care delivery for children in general practice should embrace the opportunity to learn with and from parents.

## How this fits in

Children are vulnerable to healthcare-associated harm with parents, families, guardians, and caregivers potentially playing a key role in both contributing to and mitigating against paediatric patient safety incidents. This study reports on the duality of this parental role within paediatric safety by characterising national patient safety reports relating to general practice, where parents are directly involved in the incident being described. Parents can directly influence paediatric safety within general practice, including taking positive, mitigatory steps to keep their children safe and prevent harm. Practices, researchers, and policymakers should capitalise on the role parents play in identifying and mitigating paediatric safety incidents and better incorporate the parent voice in decision making and priority setting.

## Introduction

Unintended and harmful outcomes of healthcare delivery are a frequent public health issue,^1–3^ with children being particularly vulnerable to healthcare-associated harm.^4^ Children are dependent on others to recognise illness and seek help on their behalf, having a high dependency, especially among younger children, on their parents, guardians, and informal caregivers (henceforth referred to as parents) to support their healthcare needs.^5^ Within primary care settings, Rees *et al* described the most commonly occurring patient safety incidents involving sick children, with 30% of reported incidents resulting in harm, including death.^6^

Parents therefore play a key role in the aetiology of unsafe care, and in the detection and prevention of healthcare-associated harm.^7–9^ Medication incidents within community settings are often parental in origin (such as administering the wrong dose).^8,10,11^ However, parents can act as an important resilience mechanism for supporting healthcare systems and preventing harms.^12^ This includes medication safety, where parents share relevant information and provide necessary additional scrutiny.^13^

There have been international calls for stronger collaborative partnerships with parents to improve patient safety.^14,15^ Martha’s Rule, named after 13-year-old Martha Mills who died of sepsis in 2021 after concerns about her medical care raised by her parents were not adequately addressed,^16^ is an example of a major patient safety initiative successfully aligning with these calls. This initiative gives patients and families the ability to request a rapid review by a critical care team if they are concerned that a patient’s deteriorating condition is not being appropriately responded to.^16^ Martha’s Rule is currently being implemented across hospitals in England, and having been developed with Martha’s parents (including their support with its current roll-out)^17^ recognises the need to empower patients and their families to effectively voice their concerns and be heard. Within primary care, when parents present with an unwell child they also want to be acknowledged as competent collaborators with healthcare providers.^18^

Safer paediatric care should be co-delivered with parents, but the duality of parental involvement in this context highlights that to achieve this it is important to understand the current role parents play in paediatric healthcare safety. Understanding and characterising the nature of this role will support the co-development of harm-prevention strategies to provide safer care for children within primary care.

The aim of this study was to explore how and in what circumstances parents may inadvertently contribute to or help mitigate against paediatric patient safety incidents in general practice.

## Method

### Study design

A cross-sectional study was conducted of paediatric patient safety incidents reported from general practice where parents were directly involved in either contributing to or mitigating against the incident being described.

### Data source — patient safety incidents

Incident reports involving children were received from the National Reporting and Learning System (now called the Learn from Patient Safety Events [LFPSE] service),^19^ a repository of patient safety incident reports, including categorical data and free-text information. The reports were submitted across primary and secondary healthcare settings in England and Wales between September 2014 and February 2023.

Search terms, such as ‘mum’, ‘dad’, and ‘parent’, were applied to the reports to identify incidents with potential parental involvement. Further details relating to the incident report data handling and search term sensitivity checks are described in the study’s published protocol.^20^ Only reports where the care setting of occurrence was ‘General Practice’ were included for analysis.

Two GPs (the first author [*n* = 760] and the third author [*n* = 761]) independently reviewed the reports for inclusion criteria. Reports were included for coding if they described a paediatric safety incident, defined as *‘any unintended or unexpected* [healthcare] *incident* [involving a child less than 18 years of age]* which could have, or did, lead to harm’*,^21^ with parental involvement. The term ‘parent’ was inclusive of any family member or guardian with parental responsibilities.^22^ ‘Involvement’ included any role they played in the aetiology/chronology of the incident itself, not including involvement by virtue of genetics, for example, inherited conditions (further details of the inclusion criteria are described in the study’s protocol).^20^

To assess concordance between reviewers, 20% of reports were double coded by each reviewer and Cohen’s kappa interrater reliability statistics were calculated based on the application of the inclusion criteria and whether reports involved mitigatory or contributory parental actions. A Cohen’s kappa statistic of >0.6, which indicates substantial agreement, was sought.^23^

### Data coding

The two coders were trained in applying the PatIent SAfety (PISA) classification system to code the free-text information within the included reports. The development of the PISA classification system and its application across multiple healthcare settings has previously been described.^24^ By following the recursive model of incident analysis,^25^ PISA captures the chronological sequence of events leading to a safety incident. It incorporates multiple coding frameworks with independent classes describing the incident (‘what happened?’), the contributing factors (‘why it happened?’), the resultant harm outcome, and the level of harm.

The coders determined and characterised how parents were involved in the incidents themselves, including how parents inadvertently contributed to or helped mitigate against incidents that could result in a healthcare-associated harm. Mitigating factors were defined as *‘actions or circumstances which prevent or moderate the progression of an incident toward harming the patient’* and contributory factors defined as *‘the circumstances, actions or influences which … have played a part in the origin or development of an incident or to increase the risk of an incident’*.^26^

Existing codes within the PISA framework were used to capture the contributory actions of parents relating to a safety event. Whereas, to capture the mitigatory actions and behaviours taken by parents, new mitigatory codes were iteratively developed using the constant comparative method,^27^ in keeping with the development of the original PISA framework^1^ and novel codes generated for specific healthcare contexts.^28^ Monthly meetings took place between the coders and the team, inclusive of a parent (public) contributor (the fourth author), to discuss the parental mitigatory factors identified, themes in the data, and any queries that arose during this process.

### Analysis

Using the principles of the constant comparative method,^27^ an exploratory descriptive analysis was undertaken of the coded data to identify the most frequent primary incidents, contributing factors, mitigatory actions, and levels of harm. The coded data were described and summarised using frequency tables and cross-tabulations to explore the prevalence of recurring codes and the semantic relationships between these codes. Outcomes from the monthly team meetings guided refinements of newly generated codes and emerging relationships to be further compared with the existing data. This process continued until no new insights from the data emerged and the team agreed on the coding categories and definitions developed.

## Results

**Figure 1.**
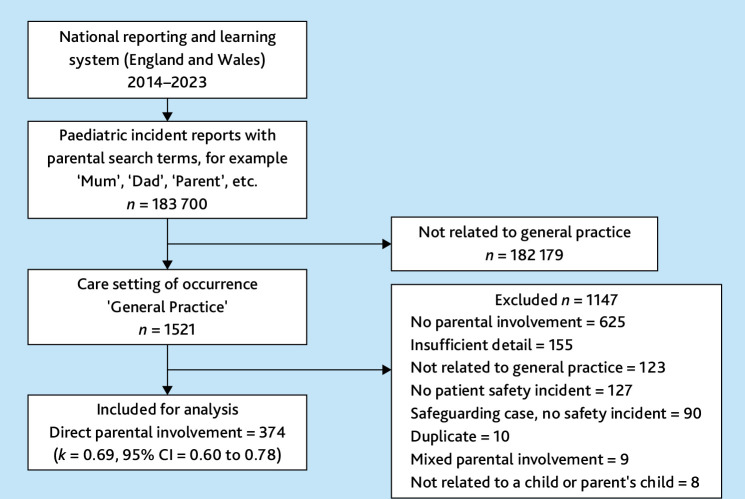
Flow diagram of sample formation.

The total number of paediatric incident reports inclusive of the parental search terms and taking place within general practice was 1521 (Figure 1). Reports were primarily excluded where there was no parental involvement in the incident (*n* = 625) or insufficient detail was provided (*n* = 155), with 374 reports included for analysis.

Most reports involved mitigatory action by parents (*n* = 287, 76.7%), with less than a quarter of reports describing a parental contributory action (*n* = 87, 23.3%). There was substantial agreement between coders determining which included reports contained mitigatory or contributory parental actions (*k* = 0.78, 95% confidence interval [CI] = 0.60 to 0.96).

Children within each age category (from ‘less than 28 days’ to ‘12 to 17 years old’) were involved in the reported incidents (see Supplementary Table S1).

### Reports with parental mitigatory involvement

Each incident report can contain ≥1 incident type. A total of 355 pooled incident types were identified for reports with parental mitigatory involvement (*n* = 287).

Parents helped mitigate a range of incident types (Table 1), most frequently involving medications (*n* = 164/355, 46.2%), specifically prescribing (*n* = 83/164, 50.6%), dispensing (*n* = 41/164, 25.0%), and vaccinations (*n* = 16/164, 9.8%). Next, they helped mitigate incidents with diagnosis and assessment (*n* = 55/355, 15.5%), administrative processes (*n* = 38/355, 10.7%), and communication issues (*n* = 38/355, 10.7%). Additional incident types are detailed in Supplementary Table S2.

**Table 1.** Safety incident categories for reports with parental mitigatory involvement^a^

Top five primary incident categories and incident types	*n *(% of incident total)	*n* (% of incident category)
Related to medications	164 (46.2)	
Prescribing medication		83 (50.6)
Dispensing medication		41 (25.0)
Vaccines, for example, administration, timeliness, or prescribing		16 (9.8)
Administration, for example, wrong dose		8 (4.9)
Errors in treatment/decision making		6 (3.7)
Medication timeliness		4 (2.4)
Prescription handling		4 (2.4)
Medication unavailable		2 (1.2)
Related to diagnosis and assessment	55 (15.5)	
Diagnosis, for example, delayed or missed		21 (38.2)
Delayed assessment		21 (38.2)
Insufficient assessment		12 (21.8)
Other		1 (1.8)
Related to administrative issues	38 (10.7)	
Managing appointments		6 (15.8)
Access to physician		19 (50.0)
Transfer of patient information		7 (18.4)
Other		6 (15.8)
Related to communication	38 (10.7)	
Between HCPs and patients/carers		20 (52.6)
Between HCPs		18 (47.4)
Related to referrals	21 (5.9)	
Decision-making process, for example, referral not done when indicated or incomplete		18 (85.7)
Administration process		3 (14.3)

^a^ *N* = 355 (total pooled incidents). Additional incident types (documentation, treatment and procedures, investigations, and other) are detailed in Supplementary Table S2. HCP = healthcare professional.

Actions to mitigate a safety incident (*n* = 304) included: recognising issues with their child’s medication (*n* = 49, 16.1%), for example, highlighting that their child was allergic to what had been prescribed; chasing healthcare appointments (*n* = 45, 14.8%), including referrals; and making complaints or providing feedback (*n* = 29, 9.5%) for organisations to learn from their concerns (see Table 2 for further examples).

**Table 2.** Summary of parental mitigatory actions (*N* = 304)

Mitigatory action category and action taken by parent	*n* (%)
Medication	153 (50.3)
Recognised issues concerning medication, for example, recognised patient was allergic before medication was given (*n* = 5)	49 (16.1)
Queried medication dose or duration	36 (11.8)
Identified issue related to vaccines, for example, recognised incorrect vaccine was administered	21 (6.9)
Chased prescription, for example, delay in receiving medication	17 (5.6)
Identified prescribing or dispensing issue	17 (5.6)
Difficulty accessing specialist medication, for example, unable to contact hospital or specialist service	6 (2.0)
Identified insufficient supply of medication	5 (1.6)
Concern regarding administration	1 (0.3)
Medication route not suitable, for example, tablet	1 (0.3)
Timely anticipated contact with a HCP	50 (16.4)
Chased healthcare appointment, for example, chased a referral (*n* = 14)	45 (14.8)
Sought private care, for example, for imaging or medication	4 (1.3)
Raised concern regarding onward referral	1 (0.3)
Complaint or feedback	29 (9.5)
Raised complaint or provided feedback on behalf of child	29 (9.5)
Diagnosis and assessment	23 (7.6)
Sought second opinion from another department or team	15 (4.9)
Re-attended for re-assessment	5 (1.6)
Queried decision making regarding management plan	3 (1.0)
Communication	15 (4.9)
Used as information source	5 (1.6)
Informed HCP that medication or treatment already given	4 (1.3)
Raised concern about poor communication	2 (0.7)
Informed HCP of specialist team input	1 (0.3)
Used as information source when documentation not complete	1 (0.3)
Informed HCP of complications faced	1 (0.3)
Chased HCP for information	1 (0.3)
Other	15 (4.9)
Parent transported child to A&E	8 (2.6)
Raised issue about environmental hazard	3 (1.0)
Raised concern about confidentiality	2 (0.7)
Parent attended walk-in clinic as GP appointment delayed	1 (0.3)
Raised concern about care environment	1 (0.3)
Investigations (for a condition)	9 (3.0)
Chased investigations, for example, requested the investigation or the result	9 (3.0)
Administrative issues	5 (1.6)
Identified administrative issue	5 (1.6)
Documentation	3 (1.0)
Highlighted documentation error	3 (1.0)
Treatment and procedures	2 (0.7)
Recognised additional care needed after first assessment	1 (0.3)
Identified care omission	1 (0.3)

A&E = accident and emergency. HCP = healthcare professional.

Reports describing parental mitigatory involvement had 199 contributory factors (Table 3). The most frequent contributing factor was a ‘mistake’ being made by a healthcare professional (*n* = 74/199, 37.2%). These mistakes were identified by a parent and their actions helped to mitigate potential harm, for example, they chased a referral that had not been sent. Where incidents related to poor ‘continuity of care’ (*n* = 35/199, 17.6%), parents chased investigation results that had not been processed and sought support from their GP for medications that were difficult to access from secondary care teams, for example, medication for epilepsy or attention deficit hyperactivity disorder. The full table of contributory factors and mitigatory parental actions is provided in Supplementary Table S3.

**Table 3.** Top two contributing factors present when parents intervened, associated parental mitigatory actions, and examples^a^

Contributing factor, parental mitigatory action category, and parental action taken	*n* (%)	Examples
Cognitive, for example, a mistake made by a HCP	74 (37.2)	
Medication/vaccine	51	
Parent queries or clarifies medication dose or duration		Highlights to GP that dose is too high
Parent chasing prescription, for example, delay in receiving or requesting alternative		Letter from secondary care not actioned and parent chasing this
Complaint	9	
Parent makes a complaint on behalf of patient		
Timely contact with HCP	7	
Parent chases healthcare appointment		Parent chasing a referral flagged that one had not been sent
Administration	3	
Parent identifies administrative issue (for example, while awaiting triage, discovers that the patient has not been correctly added to the system)		Parent identifies the wrong patient details on the prescription
Diagnosis and assessment	3	
Parent seeks a second opinion from another department or team		
Documentation	2	
Parent recognises, highlights, or resolves documentation error or incorrect details for patient		Parent queried a letter and flagged the wrong diagnosis had been recorded in the notes
Investigation	2	
Parent chases investigation for patient		Such as chasing blood results that had not been actioned
Communication	1	
Parent raises concern about poor communication		Parent told results were normal when they were abnormal
Continuity of care, for example, the delivery of a seamless service between different providers	35 (17.6)	
Medication/vaccine	19	
Difficult access to specialist medication, for example, unable to get from hospital or unable to be given by GP		Parent contacting GP for help with prescription as unable to reach a specialist service
Timely contact with HCP	8	
Parent chases healthcare appointment		Parent advocating for unwell older child not accepted by the adult secondary care team; parent requesting re-referral as child lost to follow-up
Investigation	6	
Parent chases investigation for patient		Examples include blood results that had been sent to the GP, an MRI referral that was not processed, and an X-ray referral that had been rejected but parent not informed
Communication	3	
Parent used as an information source for the healthcare team		Parent provided information from discharge letter that was not sent
Complaint	1	
Parent makes a complaint on behalf of patient		Related to difficulty accessing out-of-hours services

^a^ *N* = 199 (total contributing factors). The full table of contributory factors and mitigatory parental actions is provided in Supplementary Table S3. Contributing factors can have ≥1 parental mitigatory action. HCP = healthcare professional. MRI = magnetic resonance imaging.

Reports describing parental mitigatory actions had 415 outcomes, for example, changing the management plan or being admitted to hospital (see Supplementary Table S4). Parental mitigatory actions prevented harm from occurring in over a third of the reports (*n* = 110/287, 38.3%), and over half of the reports (*n* = 156/287, 54.4%) described how parents prevented harm (*n *= 110) or further harm (*n *= 46) from occurring.

Preventing ‘further harm’ related to incidents where a patient had already been harmed, but parental actions prevented additional harm from occurring, for example, a child was prescribed the antidepressant fluoxetine instead of the antibiotic flucloxacillin for an infection. The wrong medication was taken for a period before the parent identified the mistake. The child’s recovery was therefore delayed but the level of harm was reduced owing to their parent’s actions.

Where harm, or further harm, was prevented by parental actions, most reports related to medication incidents (*n* = 85/126, 67.5% and *n* = 28/57, 49.1%, respectively, see Supplementary Table S5), such as when a wrong medication or dose had been prescribed. Parents prevented harms from occurring across a range of incident types.

The severity of harm identified in reports with parental mitigation was primarily ‘no harm due to a mitigating action’ (*n* = 110, 38.3%), or an ‘unclear’ level of severity (*n* = 109, 38.0%). Where harm did occur, it was mostly ‘no or low harm’ (*n* = 55, 19.2%), with only a minority of reports resulting in ‘moderate or severe harm’ (*n* = 13, 4.5%).

No deaths were reported within this dataset as an outcome, but it is possible that parental actions may have prevented this outcome. For example, a child presented to the out-of-hours service and their regular GP with weight loss and polydipsia, but no investigations or follow-up were arranged. The parents took the child to the emergency department, where they were diagnosed with diabetic ketoacidosis.

### Reports with parental contributory involvement

A total of 127 pooled incidents were identified for reports with parental contributory involvement (*n* = 87). The most frequent incidents related to medications (*n* = 72/127, 56.7%), primarily concerning vaccination incidents (*n* = 43/72, 59.7%), such as the wrong vaccine being administered (*n* = 15/43, 34.9%). The ways in which parents contributed to the safety incidents are summarised in Table 4 (see Supplementary Table S6 for the full list).

**Table 4.** Safety incident categories for reports with parental contributory involvement and the parental contributory factors^a^

Top three primary incident category, top incident types, and parental contributory action	*n* (% of total incident types)	*n *(% of incident category)
**Related to medications**	72 (56.7)	
Vaccines, for example, administration (*n* = 23) or wrong vaccine (*n* = 15)		43 (59.7)
Parent did not contribute clinical details		23
Parental mistake		16
Behaviour — the way in which parent/family act or conduct themselves		5
Non-compliance — parent did not follow advice or instructions		2
Parent unable to communicate in English		1
Prescribing		11 (15.3)
Behaviour — the way in which parent/family act or conduct themselves		5
Non-compliance — parent did not follow advice or instructions		3
Parental mistake		3
Administration, for example, wrong dose		9 (12.5)
Non-compliance — parent did not follow advice or instructions		4
Parent did not contribute clinical details		3
Parental mistake		2
**Related to documentation**	25 (19.7)	
Medical records		25 (100)
Parent did not contribute clinical details		15
Parental mistake		10
Behaviour — the way in which parent/family act or conduct themselves		3
Parent unable to communicate in English		1
**Related to diagnosis and assessment**	14 (11.0)	
Delayed assessment		10 (71.4)
Non-compliance — parent did not follow advice or instructions		4
Parental mistake		3
Behaviour — the way in which parent/family act or conduct themselves		2
Parent unreachable by phone		1

^a^ *N* = 127 (total pooled incidents). The full list of incident types with associated parental contributory actions is provided in Supplementary Table S6. Each incident type can have ≥1 contributory action associated with it.

Incidents involving documentation mostly related to vaccines and the red book (a personal child health record used to record vaccinations). In nearly all cases the parent did not bring the red book, so clinical details could not be corroborated, leading to confusion and uncertainty around whether vaccines had been given.

A more specific description of each parental contributory action category, with examples, is provided in Table 5 . Most frequently, a parent did not contribute clinical details to an encounter, for example, not being aware of the child’s medical history, or a mistake was made by a parent, for example, providing incorrect information about their child’s vaccine history.

**Table 5.** Parental contributing action categories and explanations^a^

Parental contributory action and additional comments/examples	*n* (% of reports)
Parent did not contribute clinical details	28 (32.2)
Parent did not bring the red book	
Confusion around immunisations/unsure which had been given/unclear about vaccine history/did not provide information regarding whether vaccines had been given	
Parent did not provide important/relevant information about child	
Family member other than mum or dad brought child to GP (less knowledgeable of medical history)	
Unaware of when/if medication previously or recently given or any medication changes	
Parent unaware of child’s medical history	
Parental mistake	27 (31.0)
Provided own details instead of the child’s details/provided incorrect details/brought wrong child	
Parent did not seek advice in a timely manner/recognise signs of deterioration	
Parent gave unsafe medication to child	
Parent did not chase a delayed prescription	
Parent unaware of or provided incorrect information about medication/vaccination history	
Non-compliance — parent did not follow advice or instructions	20 (23.0)
Parent gave higher medication dose than prescribed	
Parent did not follow management plan or act on advice given	
Parent did not bring child to multiple appointments/book further appointment	
Parent refused treatment/vaccine	
Parent amended medication dose without seeking advice first	
Behaviour — the way in which parent/family act or conduct themselves	12 (13.8)
Parent put pressure on a healthcare professional, for example, to give vaccine already given	
Parent caused distraction	
Late for appointment/did not book appointment	
Insistent on only seeing one particular GP	
Demanding home visit for child OOH	
Parent unable to communicate in English	2 (2.3)
Language barrier created communication difficulties	
Parent unreachable by phone	1 (1.1)
Parents not contactable despite up-to-date records	
Parent had poor understanding of the system	1 (1.1)
Parent repeatedly requesting medication from GP that cannot be prescribed in primary care	

^a^ *N *= 87 (total number of reports with parental contributory involvement). OOH = out of hours.

A total of 103 outcomes were identified from the reports (see Supplementary Table S7). The most frequent outcomes were a treatment being incorrect (*n* = 29), unnecessary (*n* = 17), or delayed (*n* = 16). The severity of these outcomes was ‘unclear’ in two-thirds of the reports (*n* = 58/87, 66.7%). The remaining incidents either resulted in no harm (*n* = 20/87, 23.0%), low harm (*n* = 6/87, 6.9%), or moderate harm (*n* = 3/87, 3.4%).

## Discussion

### Summary

Parents can both mitigate and contribute to the development of a patient safety incident involving their children. This study highlights the impactful and positive mitigatory actions taken by parents to keep their children safe and how they prevented harm or further harm reaching their child in over half of those reported incidents. In addition, where a parental action contributed to a safety incident, often owing to a mistake or confusion around medication issues, these reports mostly resulted in unclear, no harm, or low harm outcomes.

The actions of parents can have a direct influence on paediatric safety within general practice, particularly where parents are able to advocate effectively on behalf of their child. The focus should shift towards viewing parents as sources of support for helping to design safer healthcare systems and identifying ways to better incorporate their insights into daily practice to strengthen patient safety efforts and reduce the risk of healthcare-associated harms.

### Strengths and limitations

The study’s systematic approach to searching a national database of incident reports, combined with the purposive sampling strategy, and structured analytic approach to making sense of incident report data has made it possible here to definitively describe the complex phenomena of how parents are involved in paediatric safety events in general practice.

Limitations to analysing incident report data are well acknowledged, including the underreporting of incidents with variable levels of detail provided within the free text.^29,30^ Given that doctors, for example, report more harm incidents than ‘near misses’,^31^ it is possible that some mitigatory actions by parents have not been captured. Although patients and the public can submit reports directly to the LFPSE service,^21^ the reports within this study’s dataset were only submitted by healthcare professionals. This study is therefore missing the patient and parent perspective in this context, and further work is being undertaken to capture their experiences.

Additional reports with direct parental involvement may have been missed, as the study’s search strategy was not encompassing of everyone with potential parental responsibility. Following sensitivity checks for terms such as ‘carer’, it was a conscious decision not to include too many search terms, as most were unlikely to identify true parental involvement.^20^

### Comparison with existing literature

National news coverage regularly spotlights the importance of the parental voice in paediatric safety, and the consequences of parents not being able to speak up effectively.^32,33^ There are several studies within secondary care calling for increased parental engagement and collaboration with healthcare professionals to further improve paediatric safety efforts,^13,14,34–36^ and the current study supports this need from a primary care perspective.

The parents within the study’s dataset were able to identify when harm did or could have occurred, reflecting studies in the hospital setting that highlighted parents’ ability to identify safety incidents^37^ and detect incidents that hospital teams otherwise missed.^38,39^

Parents are willing and prepared to engage with and support their child’s healthcare safety in the hospital setting^40^ but may require help from healthcare staff to achieve this.^41^ There is a responsibility, therefore, for the healthcare system to better enable parents to become partners in identifying issues and designing and implementing safety solutions that better incorporate the parent voice.

The current study’s data provide examples of how GPs can support parental concerns and actions to help mitigate incidents that have originated not only within primary care but also other parts of the care system, for example, where parents cannot access specialist secondary care services. GPs play a central role in the coordination of patient care in the community and between specialties.^42^ Therefore, given the complex and dynamic nature of healthcare systems,^43,44^ maintaining a functional, trusting, and collaborative relationship between parents and GPs is vital for parents to access the support they need when advocating for their child’s health. The need for further investment to streamline the interface between primary and paediatric services to support GPs when caring for children has been highlighted in a recent Royal College of Paediatrics and Child Health policy report on transforming child health services.^45^

### Implications for research and practice

Understanding how to enable parents to effectively advocate for children and prevent harms is a key priority area for research and clinical practice. However, current practice and culture within healthcare systems do not always support parental partnerships or incorporate the family experience,^46,47^ which may limit a parent’s ability to highlight safety issues like those shown in this study’s data.

Where system change is needed to ameliorate parental contributory actions or better support their mitigatory actions, rather than developing parent-focused interventions, for example, training or education, the study’s findings suggest initiatives should be less dependent on parental characteristics such as knowledge or memory. More effective alternatives to minimise mistakes and enable parents to highlight when things do go wrong may include maintaining up-to-date electronic health records, providing parents with access to children’s records, utilising electronic red books (vaccination records), and prioritising timely communication between healthcare teams, for example, discharge letters. Standardising these processes across care systems could reduce the reliance on parents to provide or chase important information relating to their child’s care.

To achieve this, it is important to move away from ‘a them and us’ culture^15^ to include the parent voice in decision making, research priority setting, intervention development, and policy changes to provide a vital lens through which to appraise safety improvements. This would take a step towards meaningful co-production with parents, ensuring their ideas, perspectives, and experiences are incorporated into system redesign processes.^48,49^

The authors plan to compare these data with other paediatric healthcare settings, to develop a greater understanding of parental involvement across multiple paediatric pathways.^20,50^

Primary care teams working to strengthen and design safer systems of care delivery for children in general practice should capitalise on the role parents play in identifying and mitigating paediatric safety incidents and embrace the opportunity to learn with and from parents.

## Data

A data-sharing agreement between Cardiff University and NHS England is in place for this study in relation to the incident report data (DSA 5236). Procedures are in place to anonymise the data sent to the study team by NHS England. All data generated, and then analysed, during this study will be available from the authors on reasonable request.
